# Supplementary material for: Bioprospection of the trichomonacidal activity of lipid extracts derived from marine macroalgae Gigartina skottsbergii
Source: PLoS One. 2023 May 8;18(5):e0285426. doi: 10.1371/journal.pone.0285426 (PMC10166524; doi:10.1371/journal.pone.0285426)
Supplement: Attachment — Submitted filename: review report of PONE-D-22-31808.docx [file pone.0285426.s002.docx]

Review report of Manuscript No. PONE-D-22-31808

“BIOPROSPECTION OF THE TRICHOMONACIDAL ACTIVITY OF LIPID EXTRACTS DERIVED AT DIFFERENT STAGES OF DEVELOPMENT FROM Gigartina skottsbergii AGAINST Trichomonas vaginalis.

Authors tried to established the trichomonacidal activity of fatty acids derived from the marine macroalgae Gigartina skottsbergii . Overall the article is interesting and can be published after some minor revisions as follows:

**General Comments:**

1. Several abbreviations are used in the text without full form. One time mention of the full form of the used abbreviations is required for general readers

2. Though overall language is good, but there is a scope of improving the standard of language. In some cases it is too wordy and unnecessary!

3. Images/ Graphs can be more attractive and colourful.

**Specific Comments**

**Title:**

It is too wordy! It can be shortened like “Bioprospection of the trichomonacidal activity of lipid extracts derived from marine macroalgae Gigartina skottsbergii”

**Abstract:**

Line 31**:** Trichomonas vaginalis should be italic.

Line 34, 39: in vitro and in silico should be italic and the pattern should be maintained all through the manuscripts

Line 34-35: ‘marine microalgae’ should be placed before ‘Gigartina skottsbergii’

**Introduction:**

Line 74**: ‘….**considered important considering …….’ Rewrite the sentence.

Line 78: Instead of ‘non-viral infections’ write ‘protozoan infection’

Line 79: Add ‘particularly in female’ after ‘genitourinary system’

**Material and Methods:**

**Macroalgae Specimens**

Line 93-99: How the specimen is identified? What is its accession no.? Necessary information should be provided

**Extraction and Derivatization**

Line 103: Why the fatty acids are only extracted? Are there any clues for using fatty acids as anti-protozoan drug? Because alga produces an array of secondary metabolites such as alkaloids, flavonoids, glycosides, terpenoids, and phenazines which have alleged medicinal properties!

Line 103: Bligh and Dyer (B&D) - year and reference no. is missing

Line 110: ‘described by [15]’- author is missing!

**Instrumentation and Quantification**

Line 130-134: ‘Methanol, chloroform, …………..purchased from Supelco (Bellefonte, USA).’ Unnecessary and delete from the text.

Line 141: Full form of ‘TvMGL, TvPNP, TvTPI, and TvLDH’ should be given

Line 145-46: ‘Linker structures ….. PubChem program’.- unnecessary and delete

Line 149,152: ‘Autodock’ should be ‘AutoDock’

**Parasite culture conditions**

Line 166: Full form of ‘GFG, GFT, and GF’ should be given

Line 166: Correct the information ‘Sena-Lopes (et al., 2017)’- et al. should be italic and outside the parenthesis, also provide the reference no. within [ x]

Line 173: ‘2.6 × 105’ should be ‘2.6 × 10^5’^

Line 180: ‘CO2’ should be ‘CO_2_’

**Cytotoxicity Assay**

Line 203: Navarrete-Vázquez (et al., 203 2015) - et al. should be italic and outside the parenthesis, also provide the reference no. within [ x]

**Gene expression analysis of T. vaginalis**

Line 237-238: ‘Forward5'-CG….AGG3'andReverse 5'-TCCT…..AAC-3'- provide space in appropriate places!

**Results:**

**Anti-T. vaginalis Activity**

Line 277: 89,61% and 86,95%, should be 89.61% and 86.95%, respectively

**Molecular docking**

Line 331: Required space is missing in ‘(∆Gbinding)’

**DISCUSSION**

Line 384: Few references are required for the comment “Several of the identified constituent compounds are already reported in the literature…”

Line 398-399: ‘Therefore, it was established that the evaluated extracts exhibited…’ this sentence is not in conformity with the previous sentence. Rewrite accordingly

Line 402: ‘Bonde (et al., 2021)’- et al. should be italic and outside the parenthesis, also provide the reference no. within [ x]

Line 448-449: ‘(LEITSCH; KOLARICH; DUCHÊNE, 2010; JONES et 449 al., 2016)’- it is not the Plos Ones’ reference style!

**Conclusion:**

Line 478: Write ‘Gigartina skottsbergii’ after ‘ subantarctic marine algae’

Line 479: ‘T. vaginalis’ should be italic
